# Editorial: Well-Being of School Teachers in Their Work Environment

**DOI:** 10.3389/fpsyg.2020.01239

**Published:** 2020-08-12

**Authors:** Paula Benevene, Simona De Stasio, Caterina Fiorilli

**Affiliations:** Department of Human Sciences, Libera Università Maria SS. Assunta University, Rome, Italy

**Keywords:** teachers' well-being, teachers' resilience, teachers' happiness, teachers' health, school's environment, workplace well-being, positive psychology

Teachers' well-being has received much attention over the past decades, in the light of the major increase in sick leave as well as job quitting among teachers across different cultures and countries. It is in fact well-known in the literature that teaching is a demanding, challenging profession, exposed to stress, burnout, and more in general, a high attrition rate. The majority of studies have in fact targeted negative indicators of teacher functioning, but more recently, following the mainstream of positive psychology, more attention has been devoted to teachers' well-being. Well-being is not just the mere absence of illness at work. Rather, it refers to healthy and successful functioning of teachers at work. In fact, while physical, psychological, and mental health refers more to the lack of impairment, well-being refers more to the ability of teachers to develop a positive though dynamic equilibrium between teachers' resources and their challenges/demands (environmental, social, individual, physical, mental, psychological).

Well-being has been found to be linked to a positive relationship with students, colleagues, and families, as well as to higher academic results of the pupils. Well-being is a multifaceted concept, comprising cognitive and affective as well as physical and mental components; it encompasses dispositional, personal, organizational, and environmental factors. Addressing protective factors may help to develop resilience and more effective ways of addressing the impact caused by negative factors of the teaching workplace. Therefore, the goal of this Research Topic was to encourage new understandings of teachers' well-being from interdisciplinary psychological perspectives.

The articles collected in this special issue cover a huge variety of approaches (both qualitative and quantitative), countries (Western such as Italy, Finland, Switzerland, and Czeck, as well as non-Western ones, such as Chile, China, Tunisia, Australia) and types of studies (empirical research, research-action, training, validation of instruments). Studies presented here vary from the training of teachers to the individual and organizational factors affecting or protecting teachers' well-being and health.

We are happy to offer such a wealth of innovative material, with the aim of not only deepening the knowledge on the subject but also offering practical ways to promote teachers' well-being.

The first group of articles addresses the relationship between burnout, the work environment, and individual characteristics. These results are important because they offer suggestions on how to foster resilience among teachers, also in the light of the management of schools and educational systems.

More specifically, a study carried out in Czech Elementary Schools (Smetackova et al.) confirmed the close relationship between burnout and low self-efficacy, poor use of positive coping strategies, and low social support from colleagues and principals. In addition, teachers with developing or developed burnout syndrome feel more often unsatisfied about their profession. These results are interesting given that the data was collected in a post-communist country like Czechia, since the knowledge about this country is still underdeveloped and, as the authors point out, every educational system, with its specific values and organizational structure, introduces different challenges, addressing different ways of tackling them.

These findings are in line with another article, presenting a comparison between two groups of primary school teachers, one Italian (Naples) and the other Swiss (Cantone Ticino) (Parrello et al.). In fact, from the authors' findings it is possible to hypothesize that different professional cultures impact differently on teachers' burnout, while macro factors of the work context have a limited impact. More in general, their study aimed at understanding if and to what extent the socio-cultural and economic differences of the two contexts, as well as dispositional, relational, and organizational variables, impact teacher burnout differently.

The research carried out among a group of Finnish school teachers adopted a person-oriented approach, which offers the possibility of identifying how teachers may endorse simultaneously contradictory measures of work well-being, such as burnout and engagement (Salmela-Aro et al.). In fact, the authors examined different kinds of teachers' profiles, based on work burnout and engagement. This study revealed for the first time a profile of teachers being engaged but at the same time at risk of exhaustion and of feeling of inadequacy. Identifying risk factors for teacher burnout is important but also understanding protective factors is necessary.

The study carried out by Fiorilli et al. investigates the relation between teachers' levels of burnout on the one hand and their emotional intelligence on the other, as well as the internal and external social support received. Internal support comes from within the work setting itself (i.e., support from colleagues, supervisors, school principals), while external support refers to the teachers' private life (i.e., support from friends, family members, partners). Findings suggest that personal competence in the emotional domain may significantly contribute in teachers' well-being. More than that, emotional intelligence has proved to be susceptible of improvement through training programs, thus preventing teacher burnout.

Again, in terms of action that can be taken in order to reduce and prevent burnout, the study from Chirico et al. offers an interesting contribution. This study deals with an experience conducted in a group of teachers of a religious institute, in which prayer was used as a technique to prevent burnout. The study begins from a fact that techniques involving higher mental functions, such as transcendental meditation, have been used in stress and burnout prevention programs. Thus, praying could be effective, no less than meditation and other spiritual or mind–body techniques, in contrasting the negative effects of occupational stress and preventing burnout among teachers and, possibly, other human service professionals (Ballantyne and Retell).

The article written by Ballantyne and Retell presents a paper on praxis shock. More precisely, the authors investigate how teachers' self-reported levels of burnout and self-efficacy, well-being, and praxis shock vary as a function of time in the profession and how the constructs of well-being, burnout, and self-efficacy relate to teachers' experiences of praxis shock. It has to be noted that no previous work has approached the experiences of teachers' praxis shock as a way to investigate the interrelationships that might exist between self-efficacy, well-being, and burnout. Their paper reveals that praxis shock may occur at multiple points in a music teachers' career and that it is linked with reported burnout, well-being, and self-efficacy.

A second group of articles addresses the issue of teachers' subjective happiness, and its relation with factors of health and well-being among teachers, such as self-esteem and positive emotions.

The study of Benevene et al. deepens the understanding of the effects of happiness and self-esteem, as dispositional traits, on the health of teachers, as well as of the role played by the working environment in generating positive affection, thus mediating between the dispositional traits and teachers' health. The study is carried out in a group of full-time in-service teachers from Italy, and its findings show that self-esteem endorses health-related behaviors. Moreover, when teachers acknowledge their workplace as a context in which they feel happy, the impact of dispositional happiness and self-esteem on health conditions is higher.

The article by De Stasio et al. deals with kindergarten teachers, which are still poorly addressed in the literature on teachers' well-being. This study highlights that subjective happiness and compassion are linked. In addition, subjective happiness and compassion are related with work engagement, through the mediation of proactive strategies, that is, self-regulation and co-regulation, meaning, respectively, the ability to identify and use resources for coping with stressors and the ability to seek and receive social support from colleagues.

So far, the relevance of offering training opportunities to teachers, following the mainstream of positive psychology (Rahm and Heise), has presented the results of a successful training aimed at fostering subjective well-being among a group of German teachers. Some of the main issues addressed in the course of the training intervention include the consequences of both positive and negative emotions, emotion regulation, time management, and gratitude. The analysis of the results of the experimental group, compared with the control group, confirmed the efficacy of the intervention.

A third group of articles collected in this special issue deals with the impact of the school context on factors affecting teachers' well-being. This is by far the largest group of articles, showing the increasing interest in the school dynamics as well as in the interaction of teachers with their workplace environment.

The article by De Carlo et al. examines the relationship between contextual work-related factors on the one hand, in terms of job demands and job resources, and work–family conflicts on the other. Building on the Job Demands-Resources (JD-R) model, authors hypothesize that job demands (that is, qualitative and quantitative workload) are positively associated with work–family conflicts among teachers. Moreover, in line with the buffer hypothesis of the JD-R, the authors explore how job resources, in terms of support from supervisors, job autonomy, and participation in decision making, have an impact on work–family conflicts.

The article by Addimando presents a study carried out in a group of in-service primary and junior schools in Switzerland, and it shows how positive working conditions leading to teachers' well-being are associated with more effective teaching practices. The author explores how teachers who perceive a supportive and satisfying working environment (both in terms of internal and external resources) are more likely to be engaged in their activities and how this in turn leads to a more heterogeneous array of teaching practices with their students.

The paper from Parrello et al. presents a research action carried out in Italy, in 12 suburban secondary schools of six Italian cities. Teachers were involved in a Teacher Participatory Action Research (T-PAR) called the “Crossing Educational Boundaries” project. The article shows how T-PAR can be a powerful form of professional development for teachers: it actively involves them, improving their awareness of themselves, of their emotions and actions, and of their empowerment. Actively engaging the teachers and working with them to plan new methods to reduce the rates of educational wastage, in turn, is a way to reinforce their resilience and help them to give purpose to their jobs, thus improving their well-being at school.

De Cordova et al. present a paper on teachers who are exposed to violent behaviors on the part of students and/or their parents. Their study addresses an increasing phenomenon faced by teachers and represents one of the most serious work-related stress factors affecting the teaching profession. This article deals with the capacity of teachers to deal with violence and to develop a more resilient mindset. In fact, teachers can experience occupational well-being even if they are subjected to aggressive behaviors: supportive leadership and good relationships with colleagues are valuable resources for fostering positive emotions and teachers' flourishing.

The paper by Barbieri et al. investigates the relationship between Italian teachers' well-being, socio-demographic characteristics and professional background. Using data from the 2015 wave of the Program for International Student Assessment (PISA), the study deepens the knowledge on how teachers' positive perception of the working environment, in terms of availability of adequate human and physical resources, and professional development opportunities, may develop a substantial level of well-being at work, and how these factors are related to teachers' job satisfaction. Moreover, the article addresses the key role of transformational leadership in promoting teacher's well-being.

Dal Corso et al. investigate the effects of perceived performance appraisal justice on teachers' well-being. Well-being factors that were considered in the study are job performance, job satisfaction, and life satisfaction. Performance management is a key factor to enhance professional development and improve teaching quality. This process is successful only if teachers perceive it as fair, clear, and effective, namely, if it is satisfying. The study contributes to better understanding the performance management process in educational settings. Furthermore, it highlights the importance of efficacy of the performance management, which is essential not only to improve individual well-being but also to enhance teaching quality.

The article presented by Cigala et al. presents the results of a training intervention on early childhood teachers. The issue addressed in the training is the management of a multi-age classroom, perceived by the teachers themselves as making teaching and learning very difficult and ineffective. The study shows that it is possible to change the teachers' perception of their own work through training intervention and to reach positive results in terms of three specific dimensions of well-being: a greater sense of belonging to the group of colleagues, a greater sense of self-efficacy, and an idea of themselves as active and meaningful participants.

The paper by Wang and Li deals with technostress among a group of teacher of high education institutions (HEI) in China. More specifically, the authors investigate the relationships between multidimensional technostress and job performance. HEI requirements related to the use of Information and Communication Technologies (ICT) and the suitability of ICT for teachers' work are found to be critical factors affecting job performance. In addition, the study develops a comparison among teachers from different grade levels, analyzing if and to what extent HEIs' management related to ICT use tend to generate a different level of technostress among different teaching levels.

Also, the paper by Kuusimäki et al. deals with technology and teachers' well-being. Their article addresses the issue of digital communication (DC) between teachers and parents in a group of Finnish teachers. In fact, most parent–teacher communication nowadays takes place on digital platforms, but not much is known about the specific role of digital communication (DC) in building parent–teacher partnerships. This paper highlights how DC can be a strong, positive factor of promoting and supporting not only the parent–teacher partnership but also teachers' well-being.

The fourth group of articles addresses how individual factors may affect teachers' well-being.

Troesch and Bauer compared first-career teachers (FCT) with second-career teachers (SCT), that is, respectively, teachers who have no other previous professional experience but teaching and teachers who became teachers after having had a previous, different professional career. Their study explores whether FCTs and SCTs differ in how they feel challenged by professional demands associated with teaching. Their study suggests that SCTs' background as career switchers might be less important for coping with specific professional demands than the existent research literature suggest; indeed, they feel nearly as challenged when starting to teach as FCTs do.

The article by Barni et al. explores the relationship between teachers' values (conservation, openness to change, self-transcendence, and self-enhancement) and their self-efficacy, assuming as a starting point the Schwartz's theory of human values. In particular, their study aimed at analyzing the extent to which these relations are moderated by teachers' controlled and autonomous motivations for teaching. Interestingly, the study suggests that the relationships between openness to change and self-efficacy on the one hand, and self-transcendence and self-efficacy on the other, varied depending on teachers' motivations. These relations were stronger when teachers perceived less external pressure and felt to be self-determined toward teaching.

The article by Buonomo et al. deals with teachers' emotions. The authors investigate both the positive and negative emotions of teachers toward, respectively, their students and their professional role. More specifically, they explored how teachers' emotions toward students would explain their self-efficacy. This paper suggests that teachers' emotions toward professional roles may generate a broader emotional context that could influence the predictive effects of their emotions toward students.

Wong presents a study carried out in the Chinese preschool setting, observing teachers' personality and their teaching practice. More specifically, the author examines how preschool teachers' personalities are related to their beliefs of developmentally appropriate practices (DAP) and thus to their teaching practices. The paper suggests that there is a combined effect of teachers' personality and work experience in adopting DAP. Moreover, extraverted and intuitive teachers tend to show higher scores in DAP beliefs and practice than introverted teachers do.

Finally, the paper by Lizana et al. deals with teachers from a quite under-represented country in the literature on teachers' well-being: Chile. The authors carried out their study among a group of teachers working in rural schools of the Valparaíso Region, investigating the relationship between their perceived quality of life and their general health condition. Their paper suggests that resources must be made available to detect early mental and chronic health conditions of rural teachers.

A final group of articles deals with instruments useful to addressing and investigating teachers' well-being, through the validation of diagnostic instruments.

The study by Reina et al. provides evidence of the validity and reliability of the Spanish translation of presents of the Validation of a Physical Education Teachers' Self-Efficacy Instrument Toward Inclusion of Students With Disabilities (Block and Weatherford, 2013). The scale has also three subscales on intellectual, physical, and visual disabilities. The value of this work lies in the fact that teachers who receive prior training on working with disabled children would be more prepared to address diversity in the classroom, which is in turn related to their perception of self-efficacy.

Chalghaf et al. present another paper devoted to Teacher of Physical Education. More specifically, their study aims to develop a preliminary “Validation of the Teacher of Physical Education Burnout Inventory” (TPEBI) in the Arabic language, in Tunisia. Presently there is no reliable psychometric tool in the Arabic language that can be used to measure the burnout level among sports and physical education teachers. The article by Chalghaf et al. thus deals with the translation of Maslach's Burnout Inventory instrument (according to its three-dimensional theoretical model: emotional exhaustion, depersonalization, and low personal accomplishment) in Arabic.

A second paper by Chalghaf et al. aims to validate the “Academic Flow Scale” (Flow 4D 16) in the Arabic language, again in Tunisia, and to test its factor structure, in terms of internal consistency/reliability, predictive validity, and sensitivity. The flow occurs when individuals are engaged in a specific activity with clear goals and high commitment, facing challenges in proportion to their skills, fully mobilizing their competencies, and dedicating their attention to the task.

A further paper by Chalghaf et al. deals with job satisfaction, as a relevant factor of well-being. The paper aims to trans-culturally adapt and validate “Teacher Job Satisfaction Scale” created by Pepe et al. (2017). This instrument has three subscales: satisfaction with colleagues, satisfaction with parents, and satisfaction with students. The authors thus developed the “Teacher of Physical Education Job Satisfaction Inventory” (TPEJSI) in the Arabic language. This scale was administered to a Tunisian population of sports and physical education teachers and analyzed according to Pepe's theoretical model.

The paper by Converso et al. develops a tool to offer support schools in making self-assessment evaluations of the internal organizational climate and teacher morale (TM). More specifically, the article deals with the Italian version of the School Organizational Health Questionnaire (SOHQ), developed by Hart et al. (2000). The original version of the SOHQ was translated from English into Italian, and it consisted of 57 Likert-type items grouped in 12 sub-dimensions: Morale, Appraisal & Recognition, Curriculum Coordination, Effective Discipline Policy, Excessive Work Demands, Goal Congruence, Participative Decision Making, Professional Growth, Professional Interaction, Role Clarity, Student Orientation, and Support Leadership.

## Author Contributions

All authors listed have made a substantial, direct and intellectual contribution to the work, and approved it for publication.

## Conflict of Interest

The authors declare that the research was conducted in the absence of any commercial or financial relationships that could be construed as a potential conflict of interest.
